# Can Dynamic Contrast-Enhanced Magnetic Resonance Imaging Combined with Texture Analysis Differentiate Malignant Glioneuronal Tumors from Other Glioblastoma?

**DOI:** 10.1155/2012/195176

**Published:** 2011-12-01

**Authors:** Pierre-Antoine Eliat, Damien Olivié, Stephan Saïkali, Béatrice Carsin, Hervé Saint-Jalmes, Jacques D. de Certaines

**Affiliations:** ^1^PRISM, IFR 140, Biogenouest, Université de Rennes 1, Campus de Villejean, 35043 Rennes, France; ^2^LTSI, INSERM U642, Université de Rennes 1, 35000 Rennes, France; ^3^Department of Radiology, CHU Rennes, 35000 Rennes, France; ^4^Department of Neuropathology, CHU Rennes, 35000 Rennes, France; ^5^Cancer Institute Eugène Marquis, 35000 Rennes, France

## Abstract

An interesting approach has been proposed to differentiate malignant glioneuronal tumors (MGNTs) as a subclass of the WHO grade III and IV malignant gliomas. MGNT histologically resemble any WHO grade III or IV glioma but have a different biological behavior, presenting a survival twice longer as WHO glioblastomas and a lower occurrence of metastases. However, neurofilament protein immunostaining was required for identification of MGNT. Using two complementary methods, dynamic contrast-enhanced magnetic resonance imaging (DCE-MRI) and texture analysis (MRI-TA) from the same acquisition process, the challenge is to *in vivo* identify MGNT and demonstrate that MRI postprocessing could contribute to a better typing and grading of glioblastoma. Results are obtained on a preliminary group of 19 patients a posteriori selected for a blind investigation of DCE T1-weighted and TA at 1.5 T. The optimal classification (0/11 misclassified MGNT) is obtained by combining the two methods, DCE-MRI and MRI-TA.

## 1. Introduction

Glioblastomas represent the majority of the glial tumors, but their phenotypic and genotypic heterogeneities are large, as attested by the appellation “glioblastoma multiforme”. Their World Health Organization (WHO) classification is mainly based on histological criteria. As the result of a consensus, the classification associates several concepts that are discussed and debated. New approaches of malignant gliomas grading are requested for treatment individualization as well as for the development of new drugs and treatment strategies. 

An interesting approach has been proposed by Saint-Anne Hospital (Paris) to differentiate malignant glioneuronal tumors (MGNTs) from WHO grade III and IV malignant gliomas: in a previous study concerning 49 patients classified as WHO grade IV, 10 have been identified as MGNT with a survival twice longer as glioblastomas and a lower occurrence of metastases. Gross total surgical resection may be curative in some cases [1–3]. Though MGNT histologically resemble any WHO grade III or IV glioma, they have a different biological behavior. Neurofilament protein (NFP) immunostaining is strictly required for identification of MGNT by pathologist [3] and has been considered as the gold standard for this study even if it is not the only one discriminant parameter taken into account by pathologists.

Compared to histology or molecular biology, magnetic resonance imaging: (i) associates a large range of complementary acquisition and postprocessing modalities as, for instance, diffusion weighted imaging (DWI) or susceptibility-weighted imaging (SWI), dynamic contrast-enhanced (DCE) MRI or texture analysis (MRI-TA) [4–8], (ii) is performed *in vivo* and then could contribute to early diagnosis and treatment followup, and (iii) allows the mapping of intra or peritumoral heterogeneity which is a highly difficult challenge when analysing biopsies or surgical pieces. Even if genomics and proteomics will probably be in a near future the gold standard for tumor subtyping, our challenge, on the basis of the example of MGNT, was to illustrate a potential important contribution from presurgical functional MRI data. 

Using two complementary methods on the same set of MR images, a static one (MRI-TA) and a dynamic one (DCE-MRI), the challenge was to *in vivo* characterize the subclass of MGNT and to demonstrate that MRI could usefully contribute to revisit the malignant glioma typing even if an early typing of these tumors do not modify the surgery strategy.

## 2. Patients and Methods

### 2.1. Patients

Nineteen patients (9 female and 10 male patients; median age, 57 years; range 40–71 years) with grade IV gliomas according to the WHO classification were selected a posteriori for this study: 8 Glioblastoma (GBM) and 11 MGNT according to the Saint-Anne classification.

### 2.2. Pathology

All patients underwent a subtotal or a gross total resection, and a single experienced neuropathologist reviewed histological specimens. The evaluated histological parameters included necrosis, vascular proliferation, mitosis, presence of giant cells and cell, density. Tumor necrosis was recorded as present when observed in at least one area in the total histological samples available. Percentage of necrosis was estimated by analysis of the totality of the paraffin-embedded tissue. Vascular proliferation was noted as low rate when endothelial cells begin confluence and as high rate when glomeruloid aspects were noted. Mitosis was analyzed in the most mitotically active tumoral area by counting number of mitosis per 10 contiguous high-power magnification fields. Cellularity was defined as low, moderate or high, according to the presence or absence of neuropil between tumor cells. For each case, perivascular lymphocytes, multinucleated giant cells, and dense reticulin network were noted when present.

Systematic immunohistochemistry study was performed for all cases on representative paraffin-embedded tumoral tissue with glial fibrillary acidic protein (GFAP) (clone 6F2) and neurofilament (clone 2F11). When at least one tumoral cell exhibited a positive labelling for neurofilament, case was considered as a MGNT, in agreement with the Saint-Anne protocol. 

As neurofilament is rather heterogeneous inducing potential diagnosis error related to sampling for pathology examination, we have considered as MGNT all tumors exhibiting at least one cell with positive labeling for neurofilament. MGNT diagnosis also includes other information but, according to [3], we have considered that this criteria could be at this time used as the gold standard for this study.

### 2.3. Dynamic MRI Acquisition and Longitudinal Relaxation Rate Measurements

2D MRI serial acquisitions were performed with a head coil on a 1.5 T imager (GE Signa, Milwaukee, USA). The quantitative MR Imaging protocol has been adapted from previously published studies [9, 10]. 2D fast multiplanar spoiled gradient echo (FMPSPGR) sequences (TR/TE = 150/5.6 ms and variable flip angle, *θ*_1_ = 10°, *θ*_2_ = 90°) were performed. Thirteen sagittal slices were acquired with a field of view of 180 mm × 240 mm, a slice thickness of 5 mm, and a 192 × 256 matrix leading to spatial resolution of 0.94 mm × 0.94 mm. Five longitudinal relaxation time (T1) calibration vials (T1 ranging from 120 ms to 2174 ms at 1.5 T and 19°C) were positioned in the coil and simultaneously imaged with the patient. For parameter extraction, regions of interest (ROI) including about 1500 pixels were manually positioned on the tumor and on the calibration vials on postcontrast images and on the corresponding part of the precontrast image. Before contrast injection, the initial longitudinal relaxation rate *R*_10_ value of each ROI was obtained with the two images acquired with the 2 different flip angles (*θ*_1_ = 10°, *θ*_2_ = 90°). After a bolus injection of a 0.1 mmol·Kg^−1^ dose of Gd-DOTA (DOTAREM, Guerbet, France), the dynamic relaxometry curves were recorded during 15 minutes. In order to increase temporal resolution, only one T_1_ weighted acquisition with a *θ*_2_ = 90° flip angle was performed for each measurement. Indeed, for small flip angles the signal is independent on *R*_1_ and does not change after gadolinium injection. For each set of images, the temporal resolution was 28 seconds.

### 2.4. Physiological Parameters Extraction from DCE-MRI

Postprocessing of DCE-MRI was done with tools developed for ImageJ (ImageJ, Rasband, W.S., US National Institutes of Health, Bethesda, MD, USA http://rsb.info.nih.gov/ij/, 1997–2011) and IDL 5.2 (Research System Incorporation, Boulder, CO, USA). After injection of a bolus *D* (mmol·kg^−1^) of Gd-DOTA, which distributes in the extracellular space, the relaxation rate of the tissue at time *t* after the injection is

(1) $$R_{1}\left(t\right)=R_{10}+rv_{e}C_{\text{EES}}\left(t\right),$$

where *r* (mmol·s^−1^) is the relaxivity of the extracellular fluid, *C*_EES_ the contrast agent concentration in the extravascular extracellular space (EES) at time *t*, *R*_10_ (s^−1^) the relaxation rate before contrast injection, and *R*_1_(*t*) (s^−1^) the relaxation rate after contrast injection.

At time *t* after contrast injection, the plasma concentration is given by the exponential relation(6)

(2) $$C_{p}\left(t\right)=D\cdot \sum_{i=1,2}a_{i}\cdot e^{-m_{i}t},$$

*a*_*i*_ and *m*_*i*_ are plasmatic constants describing the early mixing phase (*i* = 1) and the later extraction phase (*i* = 2). On the Basis of Weinmann et al. data [11], Tofts and Kermode established that for Gd-DTPA *a*_1_ = 3,99 kg·l^−1^, *a*_2_ = 4.78 kg·l^−1^, *m*_1_ = 0.144 min^−1^, and *m*_2_ = 0.011 min^−1^. By using the model described by Tofts and Kermode [12], the contrast agent concentration in EES is

(3) $$C_{\text{EES}}\left(t\right)=\frac{K^{\mathrm{trans}}}{v_{e}}\cdot \overset{}{\underset{i=1,2}{\sum }}\frac{a_{i}\cdot \left(e^{-(K^{\mathrm{trans}}/v_{e})t}-e^{-m_{i}t}\right)}{m_{i}-\left(K^{\mathrm{trans}}/v_{e}\right)},$$

*K* ^trans⁡^ (min^−1^) is the transfer constant which characterizes the blood-tissue exchange processes. From (1) and (3), we obtain

(4) $$\begin{array}{cc} \Delta R_{1}\left(t\right) & =R_{1}\left(t\right)-R_{10}\\ & =K^{\mathrm{trans}}\cdot r\cdot \overset{}{\underset{i=1,2}{\sum }}\frac{a_{i}\cdot \left(e^{-(K^{\mathrm{trans}}/v_{e})t}-e^{-m_{i}t}\right)}{m_{i}-\left(K^{\mathrm{trans}}/v_{e}\right)}, \end{array}$$

*K* ^trans⁡^ and *v*_*e*_ are calculated by fitting the experimental data on (4) using the Levenberg-Marquadt nonlinear regression method. In addition to transfer constant (*K* ^trans⁡^ min^−1^) and EES fraction *v*_*e*_, the following parameters were computed from the fitted Δ*R*_1_(*t*) curves: maximum relaxation rate (Δ*R*_1_max⁡  s^−1^), time to peak (TTP s), and slopes at 30 s (*s*30 s^−2^).

### 2.5. MRI Texture Analysis

On each tumor, one Region Of Interest (ROI) of around 1500 pixels was selected by the radiologist. Mazda imaging analysis software (Institute of Electronics, Technical University of Lodz, Poland, version 3.20 http://www.eletel.p.lodz.pl/programy/mazda/) was used for texture parameters calculation in ROIs. Three statistical methods of TA were applied giving different texture parameters: (i) gray level histogram (GLH) parameters (mean, variance, skewness, kurtosis, and percentiles) that provide statistics on signal intensity distribution, (ii) co-occurrence matrix (COM) parameters (e.g., entropy, energy, correlation, contrast, inverse difference moment, etc.); this matrix provides statistics on the joint gray-level relationships by evaluating the probability that a gray level *i* occurs, at a distance *d* and angle **θ**, from another gray level *j* in the image, thus evaluating image homogeneity, directionality and internal arrangement, and (iii) the run-length distribution matrix (RLM) parameters (gray level distribution, run-length distribution, run percentage, etc.); these parameters describe mainly the coarseness of the image. A “run” is defined as a sequence of identical gray level values in a certain direction. This method calculates the probability function *p*(*i*, *d*) in which each gray level *i* has a run of length *d*. Further details of these methods can be found in [13].

### 2.6. Statistical Analysis

The Mann-Whitney test was used for the estimation of statistical significance of imaging parameters. A *P* value less than 0.05 was considered as statistically significant. Principal component analysis (PCA) and hierarchical ascendant classification (HAC) were performed for each class of TA parameters and for parameters from DCE-MRI. Analysis were performed with Xlstat (Addinsoft, Paris, France). Positive predictive value (PPV), negative predictive value (NPV), sensitivity, and specificity have been calculated from HAC.

## 3. Results

### 3.1. Pathology

All tumors exhibited GFAP expressing cells with great variation in tumor areas. Neurofilament positive tumor cells were present in 11 cases. These tumoral cells were cytologically indistinct from other tumor cells with great variation in their number between the different cases. All tumors exhibited marked nuclear atypia and high rate of vascular proliferation. Mitosis were present in all cases with a mean of 25 mitosis/10 HPF for GBM group and 21 mitosis/10 HPF for MGNT group. Tumoral necrosis was noted in all cases with a mean of 50% for GBM group and 38% for MGNT group. Only one GBM displayed moderate cellular density (12%), other cases presenting a high cellular density. In MGNT group, 5 cases were moderately cellular (45%). Numerous multinucleated giant cells were observed in 2 GBM (25%) and in 5 MGNT (45%). Accentuation of the reticulin network was always noted and lymphocytic perivascular cuffing was seen in 2 GBM (25%) and 7 MGNT (63%) (Tables 1 and 2).

### 3.2. MRI Visual Examination

**Figure 1:**
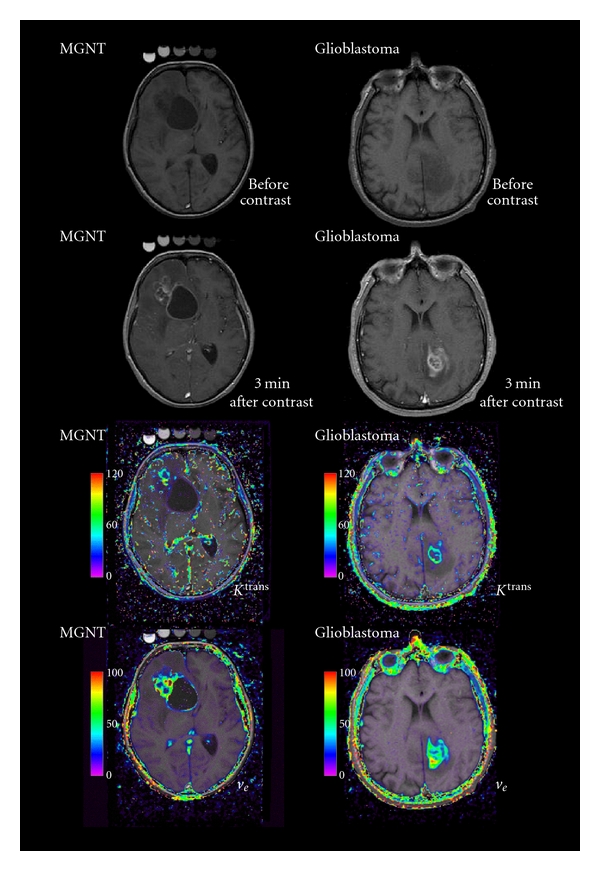
From top to bottom T1-weighted MR image before and 3 min after contrast agent injection, calculated map of transfer constant (*K* ^trans⁡^ in min^−1^) and of extracellular extravascular space fraction (*v*_*e*_ in %). An example of a malignant glioneuronal tumor is on the left and of a glioblastoma is on the right. The color code increases from purple to red. On the postcontrast T1 images the MGNT shows a complex structure, whereas the GBM sows a typical enhanced ring.

Visual examination of MR images by the neuroradiologist before and after contrast agent does not discriminate MGNT. The two expected MRI morphological signs for MGNT characterization, meningotropism in MGNT and ring structure in other WHO glioblastomas, are not significant (Tables 1 and 2). However, Figure 1 shows typical well delimited ring pattern of GBM and a more complex “grape-like” appearance of the presented MGNT.

### 3.3. DCE-MRI

Parameters describing enhancement curve and physiological parameters have been calculated for each patient (Tables 1 and 2). These parameters can be presented as physiological maps (Figure 1).

### 3.4. Texture Analysis

Parameters from the GLH, COM, and RLM were extracted from postinjection T1 weighted images (Tables 1 and 2).

### 3.5. Statistical Analysis

Using independently the two methods, MRI-TA and DCE-MRI, a rather poor discrimination between GBM and MGNT is obtained. Although higher values of *K* ^trans⁡^ and s30 are observed for MGNT, there are no statistical significant differences for DCE-MRI related parameters. From the best selected TA features horizontal run length nonuniformity (hRLN) and horizontal grey level nonuniformity (hGLN) from the run length matrix method present a statistical significant difference between MGNT and GBM with *P* values, respectively, *P* = 0.008 and *P* = 0.017. When associating both MRI-TA and DCE-MRI, 45% of the information is given by the 3 first factorial axis which are mainly weighted by hRLN, hGLN and by angular second moment (ASM), sum entropy and entropy from the cooccurrence matrix method. 11/11 correct classification of MGNT is obtained (Table 3) with a positive predictive value of 79%, a negative predictive value of 100%, a sensitivity of 100%, and a specificity of 62%.

## 4. Discussion

Though DCE-MRI has already been used in neuro-oncology, previous results have mainly concerned low versus high WHO grades [14–16] and have not revisited the WHO grading of malignant gliomas. Also, MRI texture analysis has opened exciting perspectives in the evaluation of intra-, peri-, and extratumoral heterogeneity but has not been already oriented to an attempt of malignant gliomas subgrading [4, 5].

This study associating DCE and TA is, at our knowledge, the first attempt to *in vivo* indentify the subclass of MGNT from glioblastoma. Associating DCE-MRI and MRI-TA, a sensitivity of 100% and a specificity of 62% have been obtained in the limited population already studied. The rather low specificity may be improved by adding texture parameters from T2 and/or diffusion weighted images as well as by 3D TA approaches. This preliminary result suggests that *in vivo* MRI associated with appropriate postprocessing methods could characterize MGNT up to now identified by *ex vivo* immunostaining histology. One important parameter in pathology typing of MGNT is the angiogenic status, well expressed by the *K* ^trans⁡^ parameter in DCE MRI. It has also to be noticed that the relevant MRI-TA parameters are second order parameters, then not detected by the visual observation of the radiologist who only can detect first order texture. Concerning other WHO high-grade gliomas, it has to be noticed a large heterogeneity concerning these DCE-MRI and MRI-TA parameters, suggesting again the clinical interest of tumor individualization.

## 5. Conclusion

These preliminary results show the potential interest of DCE-MRI and MRI-TA association for *in vivo* tumor characterization, an exciting challenge for subtyping of glioblastoma. Furthermore, if associated with molecular tumor characterization by genomics or proteomics, functional imaging has the potential to provide additional information on tumor heterogeneity, a highly relevant parameter for tumor grading.

## Figures and Tables

**Table 1:** MRI and pathology data (pathology).

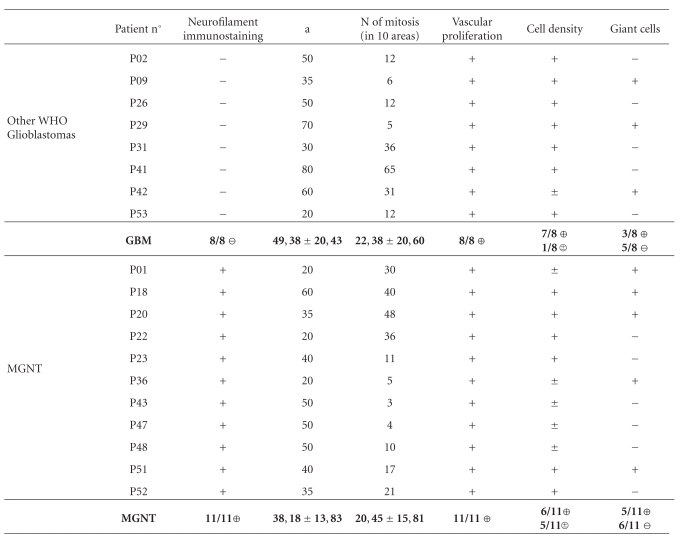

**Table 2:** MRI and pathology data (magnetic resonance imaging).

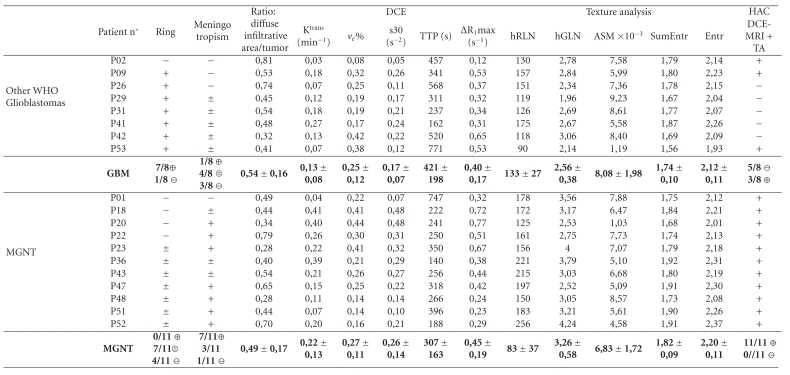

**Table 3:** Positive predictive value, negative predictive value, sensitivity and specificity (in %) obtained from hierarchical ascendant classification of both MRI-texture analysis run length matrix parameters (RLM) and co-occurrence matrix parameters (COM) and dynamic contrast enhancement MRI parameters for *in vivo* discrimination of MGNT from glioblastoma type.

	Positive predictive value	Negative predictive value	Sensitivity	Specificity
MRI-TA RLM	58	43	64	37
MRI-TA COM	75	71	82	62
DCE-MRI	71	80	64	50
MRI-TA COM + DCE-MRI	79	100	100	62

## References

[1] Daumas-Duport C, Beuvon F, Varlet P, Fallet-Bianco C (2000). Gliomas : WHO and Sainte-Anne Hospital classifications. *Annales de Pathologie*.

[2] Varlet P, Jouvet A, Miquel C, Saint-Pierre G, Beuvon F, Daumas-Duport C (2005). Criteria of diagnosis and grading of oligodendrogliomas or oligo-astrocytomas according to the WHO and Sainte-Anne classifications. *Neurochirurgie*.

[3] Varlet P, Soni D, Miquel C (2004). New variants of malignant glioneuronal tumors: a clinicopathological study of 40 cases. *Neurosurgery*.

[4] Herlidou-Même S, Constans JM, Carsin B (2003). MRI texture analysis on texture test objects, normal brain and intracranial tumors. *Magnetic Resonance Imaging*.

[5] Mahmoud-Ghoneim D, Toussaint G, Constans JM, de Certaines JD (2003). Three dimensional texture analysis in MRI: a preliminary evaluation in gliomas. *Magnetic Resonance Imaging*.

[6] Provenzale JM, Mukundan S, Barboriak DP (2006). Diffusion-weighted and perfusion MR imaging for brain tumor characterization and assessment of treatment response. *Radiology*.

[7] Rauscher A, Sedlacik J, Barth M, Haacke EM, Reichenbach JR (2005). Nonnvasive assessment of vascular architecture and function during modulated blood oxygenation using susceptibility weighted magnetic resonance imaging. *Magnetic Resonance in Medicine*.

[8] Sehgal V, Delproposto Z, Haacke EM (2005). Clinical applications of neuroimaging with susceptibility-weighted imaging. *Journal of Magnetic Resonance Imaging*.

[9] Eliat PA, Dedieu V, Bertino C (2004). Magnetic resonance imaging contrast-enhanced relaxometry of breast tumors: an MRI multicenter investigation concerning 100 patients. *Magnetic Resonance Imaging*.

[10] Vincensini D, Dedieu V, Renou JP, Otal P, Joffre F (2003). Measurements of extracellular volume fraction and capillary permeability in tissues using dynamic spin-lattice relaxometry: studies in rabbit muscles. *Magnetic Resonance Imaging*.

[11] Weinmann HJ, Laniado M, Mützel W (1984). Pharmacokinetics of GdDTPA/dimeglumine after intravenous injection into healthy volunteers. *Physiological Chemistry and Physics and Medical NMR*.

[12] Tofts PS, Kermode AG (1991). Measurement of the blood-brain barrier permeability and leakage space using dynamic MR imaging. 1. Fundamental concepts. *Magnetic Resonance in Medicine*.

[13] Petrou M, García Sevilla P (2006). *Image Processing: Dealing With Texture*.

[14] Lüdemann L, Grieger W, Wurm R, Budzisch M, Hamm B, Zimmer C (2001). Comparison of dynamic contrast-enhanced MRI with WHO tumor grading for gliomas. *European Radiology*.

[15] Lüdemann L, Hamm B, Zimmer C (2000). Pharmacokinetic analysis of glioma compartments with dynamic Gd-DTPA-enhanced magnetic resonance imaging. *Magnetic Resonance Imaging*.

[16] Roberts HC, Roberts TP, Bollen AW, Ley S, Brasch RC, Dillon WP (2001). Correlation of microvascular permeability derived from dynamic contrast-enhanced MR imaging with histologic grade and tumor labeling index: a study in human brain tumors. *Academic Radiology*.

